# Ageing across borders: psychological wellbeing, health and healthcare access among Latvian migrants in Europe

**DOI:** 10.3389/fpsyg.2026.1897187

**Published:** 2026-09-23

**Authors:** Ieva Reine, Ilze Koroļeva, Aleksandrs Aleksandrovs, Inta Mieriņa

**Affiliations:** 1 Department of Public Health and Caring Sciences, Uppsala University, Uppsala, Sweden; 2 Statistics Unit, Rīga Stradiņš University, Riga, Latvia; 3 Faculty of Humanities, Institute of Philosophy and Sociology, University of Latvia, Riga, Latvia

**Keywords:** active ageing, Europe, healthcare access, Latvian diaspora, migration cohort, older migrants, physical health, psychological wellbeing

## Abstract

**Background:**

Population ageing is increasingly occurring in societies shaped by migration. Older migrants differ substantially in migration cohort, duration of residence, labour-market histories, language proficiency, pension rights, social networks and access to welfare institutions. For ageing migrants, healthcare access is not only a service-use outcome but also an indicator of welfare anchorage, institutional trust and the practical capacity to translate formal rights into effective care.

**Objective:**

This study examines health outcomes and healthcare-access patterns among Latvian migrants aged 45 years and older. It asks whether mobility pattern is associated with poor psychological and physical health, and whether healthcare use is organised locally, transnationally or across multiple countries.

**Methods:**

The analysis draws on the 2019 wave of the Latvian emigrant survey. The analytical focus is on respondents aged 45+. Mobility pattern/place of residence was classified as living outside Latvia, living mostly outside Latvia, or alternating between Latvia and another country. Health outcomes included self-rated general health, poor physical health days in the past 30 days, poor psychological health days in the past 30 days, and frequent mental distress. Healthcare access was measured by whether respondents had received healthcare in Latvia, in the country of residence, or in another country during the previous 5 years. Descriptive statistics, cross-tabulations, ANOVA and regression-oriented interpretation were used to examine health outcomes and healthcare-access location.

**Results:**

Poor psychological health days did not differ significantly across mobility groups, *F*(2, 3,322) = 1.472, *p* = 0.230. Poor physical health days also did not differ significantly, *F*(2, 3,400) = 1.667, *p* = 0.189. In contrast, healthcare-access location differed strongly by mobility pattern. Healthcare use in Latvia increased from 34.8% among respondents living outside Latvia to 55.5% among those living mostly outside Latvia and 72.6% among those alternating between Latvia and another country. Healthcare use in the country of residence decreased across the same mobility gradient, from 87.2 to 76.2 and 58.7%, respectively. Use of healthcare in another country was highest among alternating migrants, at 18.0%.

**Conclusion:**

Among ageing Latvian migrants, mobility pattern does not significantly differentiate recent poor physical or psychological health days, but it strongly differentiates where healthcare is obtained. The findings suggest that later-life healthcare use among migrants reflects not only morbidity, but also language, institutional familiarity, trust, welfare anchorage and transnational mobility. Active ageing among migrants therefore depends not only on health status, but also on effective, understandable and continuous access to healthcare across borders.

## 1 Introduction

Population ageing is increasingly unfolding in societies shaped by international migration. Across Europe, older populations now include long-established diaspora groups, post-war labour migrants, refugees, postcolonial minorities, return migrants, retirement migrants and more recent intra-European mobile citizens. These groups differ markedly in duration of residence, legal and citizenship status, labour-market histories, host-country language proficiency, pension rights, family networks and practical capacity to access welfare institutions. Consequently, the health and wellbeing of older migrants cannot be adequately understood through chronological age alone. It requires a life-course perspective that considers when migration occurred, under what conditions, how migrants became anchored in destination societies, and which resources enable or constrain health, participation, security and autonomy in later life.

The WHO active ageing framework defines active ageing as the optimisation of opportunities for health, participation and security in order to enhance quality of life as people age (World Health Organization [WHO], 2002). Applied to migrant populations, this framework draws attention to the social and institutional conditions that make active ageing possible. Older migrants’ health outcomes and healthcare access are shaped not only by morbidity, but also by socioeconomic position, host-country language proficiency, health literacy, institutional trust, legal entitlements, pension security, social support, discrimination and transnational ties. Healthcare access is therefore both a health-services outcome and a broader marker of welfare anchorage: it indicates whether older migrants can translate formal rights into effective, timely and understandable care.

Research on ageing and migration increasingly emphasises that older migrants should not be treated as a homogeneous vulnerable group. Rather, later-life vulnerability and agency are produced through intersections between age, migration status, gender, class, ethnicity, language, family position and welfare-state context (Ciobanu et al., 2017; Smarika et al., 2023). Some older migrants may be strongly embedded in the healthcare and pension systems of their country of residence, while others may continue to rely on the country of origin or on several national systems. This distinction is particularly important in Europe, where freedom of movement, uneven welfare systems, cross-border family lives and post-socialist labour mobility have created new forms of transnational ageing.

Healthcare access is central to this ageing-migration nexus. Previous European research has shown that migrants may face barriers related to language, unfamiliarity with healthcare systems, administrative complexity, discrimination, costs, lack of culturally appropriate services, and difficulties in communicating symptoms and treatment expectations (Mladovsky et al., 2012; Priebe et al., 2011). Among older migrants, these barriers may be intensified by chronic illness, reduced mobility, digital exclusion, cognitive decline, dependence on family members for interpretation, and uncertainty about entitlements. At the same time, older migrants may exercise agency by seeking care transnationally, drawing on trusted doctors, familiar language environments, family support, or more affordable services in the country of origin. Transnational healthcare use should therefore not be interpreted only as a sign of exclusion; it may also represent a strategy for maintaining control, continuity and security in later life.

The Latvian case is particularly relevant for examining these processes. Latvian migration has been shaped by distinct historical waves. Earlier diaspora cohorts, including people who left before or during the Second World War and their descendants, are often relatively established in host countries. Many have spent most of their lives abroad, developed host-country language competence, accumulated local pension and healthcare rights, and built social networks in the country of residence. For these groups, ageing abroad may resemble long-term diaspora or minority ageing.

In contrast, many migrants who left after the restoration of Latvian independence in 1991, and especially after Latvia’s accession to the European Union in 2004, followed more fluid labour, family, educational and circular mobility trajectories. Those now approaching or entering older age may have fragmented employment histories, divided pension contributions, partial host-country language proficiency, and continued reliance on Latvia for healthcare, family support, administrative guidance and cultural continuity. For these migrants, ageing may occur not within one welfare system, but across several institutional settings. Their healthcare practises may therefore reveal how active ageing is assembled across borders.

Similar patterns are visible elsewhere in Europe. Older Russian-speaking migrants in Finland use transnational healthcare in ways shaped by digital information access, social integration and perceived discrimination in destination-country health services (Shin et al., 2022). Post-socialist migrants approaching retirement may use migration itself as a strategy for maintaining income, work participation and security in later life (Lulle, 2020). Ageing care workers in Spain illustrate how migration, care and ageing are interconnected across borders, with older migrants simultaneously positioned as workers, caregivers, family members and future care recipients (Moré, 2021). Recent research from Italy further shows that older migrants’ return intentions are closely linked to health, care arrangements and the perceived usability of healthcare in both origin and destination countries (Çela et al., 2025). These examples demonstrate that later-life wellbeing among migrants is shaped by the interaction between health status, migration cohort, welfare anchorage, language, social integration and healthcare-system usability.

The present article examines these issues using data from Latvian migrants aged 45 years and older. It focuses on two related but analytically distinct dimensions: health outcomes and healthcare-access location. Health outcomes are measured through self-rated health, poor physical health days, poor psychological health days and frequent mental distress. Healthcare access is measured by whether respondents received healthcare in Latvia, in the country of residence, or in another country during the previous 5 years. By linking physical and psychological health with the location of healthcare use, the study asks whether mobility pattern identifies differences in health burden, or whether it primarily reflects different strategies for accessing care across borders.

The article contributes to research on active ageing, migration and health in three ways. First, it places Latvian migrants within a broader European discussion on ageing populations and transnational welfare use. Second, it distinguishes health outcomes from healthcare-access strategies, showing why similar health burdens may coexist with different care-seeking patterns. Third, it highlights the importance of migration cohort, language, mobility and welfare anchorage for understanding active ageing among older migrants. The central argument is that active ageing among migrants depends not only on whether older people are healthy, but also on whether they can secure understandable, continuous and accessible healthcare as they age across borders.

## 2 Materials and methods

### 2.1 Study design and data source

This study uses data from the 2019 wave of the Latvian emigrant survey, a large-scale survey designed to examine migration trajectories, transnational ties, sociodemographic characteristics, health, wellbeing and healthcare use among persons of Latvian origin living outside Latvia or maintaining transnational mobility patterns. The survey builds on earlier Latvian diaspora research and provides a rare empirical basis for analysing health and healthcare access among ageing migrants from a Central and Eastern European country (Kaša and Mieriņa, 2019; Reine et al., 2022).

The present analysis focuses on respondents aged 45 years and older. This age threshold was selected to capture a broader ageing trajectory, including later working life, transition to retirement and older age. In migration research, this is particularly relevant because health vulnerabilities, fragmented welfare biographies and barriers to healthcare access may emerge before statutory retirement age, especially among migrants with precarious employment histories, physically demanding work, limited host-country language proficiency or transnational family obligations. Similar approaches are used in European ageing-migration research, where older migrants are increasingly analysed through life-course, intersectional and active-ageing frameworks rather than chronological age alone (Ciobanu et al., 2017; Smarika et al., 2023; Lulle, 2020; Warnes et al., 2004).

### 2.2 Analytical sample

The analytical sample consists of Latvian migrants aged 45 years and older with valid information on current mobility pattern/place of residence and at least one of the study outcomes. Respondents were classified according to their current residence and mobility pattern into three groups: those living outside Latvia, those living mostly outside Latvia, and those alternating between Latvia and another country. This classification captures different degrees of settlement, transnational mobility and welfare-state anchorage.

The distinction is important for the study of active ageing because older migrants’ access to health-promoting resources may differ depending on whether they are primarily settled in one country or organise their everyday life, healthcare and social protection across national borders. Previous research on transnational ageing and post-socialist mobility has shown that mobility in later life may function both as a resource and as a source of vulnerability, particularly where income security, healthcare access and family responsibilities are distributed across countries (Lulle, 2020; Moré, 2021; Shin et al., 2022).

### 2.3 Measures

The principal exposure was current mobility pattern/place of residence, measured as: (1) living outside Latvia, (2) living mostly outside Latvia, or (3) alternating between Latvia and another country. This variable was used to capture different forms of settlement and transnational mobility among ageing migrants.

Health outcomes included self-rated general health, poor physical health days in the past 30 days, poor psychological health days in the past 30 days, and frequent mental distress. Self-rated general health was measured using ordered response categories ranging from very good to very bad. Poor physical health days and poor psychological health days were measured as count variables indicating the number of days during the past 30 days when respondents experienced poor physical or psychological health. Frequent mental distress was defined as 14 or more poor psychological-health days in the past 30 days, a commonly used threshold for identifying elevated mental-health burden and reduced wellbeing.

Healthcare access was measured by whether respondents had received healthcare in Latvia, in their country of residence, or in another country during the previous 5 years. These three outcomes were analysed separately because they represent different dimensions of healthcare access among transnational migrants. Healthcare use in Latvia was interpreted as an indicator of continued reliance on country-of-origin healthcare. Healthcare use in the country of residence was interpreted as an indicator of local health-system access and welfare anchorage. Healthcare use in another country was interpreted as an indicator of multi-sited or third-country healthcare strategies.

Covariates included gender, age group, education, host-country language proficiency, main language, country group and healthcare-use variables. These variables were selected because prior research shows that health and healthcare access among older migrants are shaped by social position, language, welfare entitlements, migration history and institutional familiarity (Anantapong et al., 2024; Mladovsky et al., 2012; Priebe et al., 2011; Reine et al., 2022; Shin et al., 2022; Table 1).

**Table 1:** Empirical health and healthcare-access variables used in the article.

Domain	Variable	Operationalisation	Interpretive role
Physical health	Self-rated general health	Very good, good, fair, bad, very bad	Global indicator of later-life health and active-ageing capacity
Physical health	Poor physical health days	Number of poor physical-health days in the past 30 days	Short-term physical morbidity burden
Psychological wellbeing	Poor psychological health days	Number of poor psychological-health days in the past 30 days	Short-term distress, stress or anxiety burden
Psychological wellbeing	Frequent mental distress	14 or more poor psychological-health days in the past 30 days	Indicator of elevated psychological vulnerability
Healthcare access	Healthcare in Latvia	Received healthcare in Latvia in the past 5 years: yes/no	Indicator of transnational reliance on country-of-origin care
Healthcare access	Healthcare in country of residence	Received healthcare in the country of residence in the past 5 years: yes/no	Indicator of local welfare anchorage and health-system access
Healthcare access	Healthcare in another country	Received healthcare in a third country in the past 5 years: yes/no	Indicator of multi-sited healthcare strategies

### 2.4 Statistical analysis

Descriptive statistics were used to summarise health outcomes, psychological wellbeing and healthcare access by mobility pattern and age group. Cross-tabulations were used for categorical variables, including self-rated health, frequent mental distress and location of healthcare use. Differences between groups were assessed using chi-square tests where appropriate.

Analysis of variance was used to compare mean numbers of poor psychological health days and poor physical health days across the three mobility-pattern groups. These models assessed whether current place of residence or transnational mobility pattern was associated with short-term psychological or physical health burden.

Logistic-regression analysis was used to structure the interpretation of healthcare access and health outcomes. Regression-oriented interpretation focused on whether mobility pattern was associated with healthcare use in Latvia, healthcare use in the country of residence and healthcare use in another country, while adjusting for gender, age, education, host-country language proficiency, main language, country group and healthcare-use variables. These covariates were included to account for individual and contextual factors that are known to influence migrant healthcare access and active ageing opportunities.

All analyses were conducted using IBM SPSS Statistics. Results are reported as percentages, means, F-statistics, odds ratios, 95% confidence intervals and *p*-values, depending on the type of analysis.

### 2.5 Ethical considerations

All procedures contributing to this work comply with the ethical standards of the national and institutional committees on human experimentation and with the Helsinki Declaration of 1975, as revised in 2013. The study received ethical approval from the Ethics Committee for Humanities and Social Sciences at the University of Latvia. Participants gave informed consent prior to participation. In line with the national and EU legal framework on data protection, respondents were informed about how their data would be processed, stored, and protected. Data were pseudonymised, securely stored, and separated from personal identifiers. Access rights were managed according to institutional data governance policies, and encrypted end-to-end protocols were used during data collection. The panel data linkage between survey waves was carried out only for those who had explicitly consented. These data protection measures ensured full compliance with GDPR and established best practises for longitudinal survey research.

## 3 Results

### 3.1 Health outcomes and mobility pattern

The first set of analyses examined whether short-term psychological and physical health burden differed by current mobility pattern/place of residence among Latvian migrants aged 45 years and older. The ANOVA results showed no statistically significant differences between the three mobility groups for either health outcome. Poor psychological health days in the past 30 days did not differ significantly by current place of residence, *F*(2, 3,322) = 1.472, *p* = 0.230. Poor physical health days in the past 30 days also did not differ significantly, *F*(2, 3,400) = 1.667, *p* = 0.189.

This finding is important for the interpretation of healthcare access. It suggests that differences in the location where migrants receive healthcare should not be explained simply by one mobility group being substantially less healthy than another. Instead, the findings point toward a distinction between health need and healthcare-access strategy. Ageing migrants across mobility patterns report comparable levels of recent psychological and physical health burden, but they differ in where they obtain care. This distinction is central to an active-ageing interpretation, because active ageing depends not only on health status itself, but also on the ability to translate health needs into timely, understandable and continuous care (Table 2).

**Table 2:** ANOVA results for poor psychological and physical health days by current place of residence.

Outcome	Source	Sum of squares	df	Mean square	*F*	Sig.
Poor psychological health days in past 30 days	Between groups	132.790	2	66.399	1.472	0.230
	Within groups	149,896.976	3,322	45.123		
	Total	150,029.774	3,324			
Poor physical health days in past 30 days	Between groups	92.389	2	46.195	1.667	0.189
	Within groups	94,216.090	3,400	27.711		
	Total	94,308.479	3,402			

The absence of statistically significant differences in poor psychological and physical health days does not imply that health and wellbeing are unimportant in this population. Rather, it indicates that mobility pattern alone is not sufficient to identify a clearly worse-off health group. This is consistent with recent European ageing-migration research, which emphasises that vulnerability among older migrants is intersectional and may be structured by language, social position, discrimination, institutional familiarity, income security, social isolation and migration cohort rather than by residence status alone (Ciobanu et al., 2017; Smarika et al., 2023; Reine et al., 2022). Older migrants’ mental health is also shaped by reduced social networks, physical health problems and weaker access to support systems, making it necessary to interpret psychological wellbeing within a broader life-course and social-protection framework (Anantapong et al., 2024; Table 3).

**Table 3:** Interpretation of health-outcome ANOVA results for the ageing-migration argument.

Finding	Statistical result	Substantive interpretation for active ageing
Poor psychological health days did not differ significantly by current place of residence	*F*(2, 3,322) = 1.472, *p* = 0.230	Mobility pattern alone does not identify a psychologically worse-off group. Psychosocial vulnerability may instead be patterned by gender, age, language proficiency, economic security, discrimination, family separation or social isolation.
Poor physical health days did not differ significantly by current place of residence	*F*(2, 3,400) = 1.667, *p* = 0.189	Physical health burden does not explain the strong differences in healthcare-location use. Access, navigation, language and institutional-trust factors should therefore be foregrounded.
Healthcare-use location differs strongly by mobility pattern	See Table 4	The key empirical contrast is not only health status, but where care is obtained and how ageing migrants assemble healthcare across borders.

### 3.2 Healthcare access and transnational care

Healthcare access differed sharply by mobility pattern. Migrants living outside Latvia predominantly reported using healthcare in their country of residence, whereas respondents maintaining stronger mobility ties with Latvia were substantially more likely to use healthcare in Latvia. Among those living outside Latvia, 34.8% reported receiving healthcare in Latvia during the past 5 years, compared with 55.5% among those living mostly outside Latvia and 72.6% among those alternating between Latvia and another country. In the opposite direction, healthcare use in the country of residence decreased across the mobility gradient, from 87.2% among those living outside Latvia to 76.2% among those living mostly outside Latvia and 58.7% among those alternating between Latvia and another country.

This pattern shows that ageing migrants do not necessarily access healthcare through one national system only. Those with stronger transnational mobility ties appear to assemble care through cross-border strategies, combining care in Latvia, the country of residence and, in some cases, another country. Use of healthcare in another country was highest among those alternating between Latvia and another country, at 18.0%, compared with 10.3% among those living outside Latvia and 9.3% among those living mostly outside Latvia. Group differences were statistically significant for all three healthcare-access locations (*p* < 0.005; Table 4).

**Table 4:** Healthcare access by mobility pattern among Latvian migrants.

Mobility pattern	Healthcare in Latvia: yes/no	Healthcare in country of residence: yes/no	Healthcare in another country: yes/no
Outside Latvia	34.8%/65.2%	87.2%/12.8%	10.3%/89.7%
Mostly outside Latvia	55.5%/44.5%	76.2%/23.8%	9.3%/90.7%
Alternating between Latvia and another country	72.6%/27.4%	58.7%/41.3%	18.0%/82.0%
Group difference	*p* < 0.005	*p* < 0.005	*p* < 0.005

These findings suggest that healthcare use among ageing Latvian migrants is not simply a reflection of health need, but also of access, anchorage and transnational capability. Healthcare use in the country of residence may indicate stronger local welfare-state integration and familiarity with host-country institutions. By contrast, healthcare use in Latvia may indicate continuing trust in Latvian medical professionals, linguistic familiarity, perceived affordability, easier access to certain services, or unresolved barriers in the country of residence. Use of care in a third country points to even more complex, multi-sited healthcare strategies.

The pattern is consistent with European research on transnational healthcare among older migrants. Studies of older Russian-speaking migrants in Finland show that transnational healthcare use is linked to social integration, perceived discrimination and digital access to health-related information (Shin et al., 2022). Research on post-socialist ageing and mobility also indicates that migration may become part of later-life strategies for maintaining income security, care access and active ageing rather than simply a one-time move followed by settlement (Lulle, 2020). In this sense, the Latvian case reflects a broader European pattern in which ageing, mobility and welfare-state access are increasingly distributed across borders.

### 3.3 Linking health outcomes and healthcare access

The combined results show a clear empirical sequence. First, poor psychological and physical health days did not differ significantly by mobility pattern. Second, healthcare-access location differed substantially and significantly by mobility pattern. This means that the central empirical finding is not that one group is clearly less healthy, but that groups with broadly comparable short-term health burden access care in different places. This distinction strengthens the active-ageing argument: equal or similar health burden does not necessarily mean equal access to care (Table 5).

**Table 5:** Coherent empirical story linking health outcomes and healthcare access.

Analytical step	Evidence from the data analysis	Role in the scientific argument
Health-status comparison	ANOVA showed no significant differences in poor psychological or physical health days by current place of residence	Health needs are present across groups; mobility pattern does not simply identify a sicker group.
Access-location comparison	Healthcare in Latvia increases from 34.8% among those living outside Latvia to 72.6% among those alternating between Latvia and another country	Cross-border care is central to the experience of ageing migrants with continued mobility ties.
Local anchorage comparison	Healthcare in country of residence decreases from 87.2 to 58.7% across the same mobility gradient	Lower host-country care use among mobile groups may reflect weaker local anchorage, language barriers, administrative complexity or preference/trust in Latvian care.
Multi-sited care	Use of another country is highest among alternating migrants, 18.0%	Some ageing migrants manage care across more than two national systems.
Active-ageing interpretation	Health outcomes plus access-location data	Active ageing depends on effective access, continuity and security, not only on formal entitlement.

These results also clarify the role of healthcare access as an indicator of social protection. In later life, healthcare is not only a service but also a central component of security, autonomy and participation. If older migrants must move between countries to obtain care, this may demonstrate agency and transnational resourcefulness. However, it may also indicate that formal entitlements in the country of residence are not fully translated into effective access. This is particularly relevant for migrants with weaker host-country language proficiency, limited digital skills, fragmented work histories or uncertainty about social rights.

### 3.4 Regression-oriented interpretation

The analyses also support a regression-oriented analytical strategy linking health outcomes, mobility pattern and healthcare access. The models should be interpreted as testing whether mobility and access factors remain associated with health and healthcare-use outcomes after adjustment for sociodemographic and contextual variables. The key covariates are gender, age group, education, host-country language proficiency, main language, country group and healthcare-use location (Tables 6, 7).

**Table 6:** Recommended health-outcome and healthcare-access regression models to report from the SPSS output.

Model	Dependent variable	Key predictors	Purpose
Model A	Poor/fair self-rated health vs. good/very good	Mobility pattern, gender, age, education, language, country group, healthcare-use location	Tests whether physical health vulnerability is independently associated with migration and access factors.
Model B	Any poor psychological health days vs. none	Mobility pattern, gender, age, education, language, country group, physical health, healthcare-use location	Tests whether psychological wellbeing is linked to mobility and healthcare access after accounting for health status.
Model C	Healthcare in Latvia	Mobility pattern, age, gender, education, language, country group, health outcomes	Tests whether reliance on Latvia is explained by mobility and access barriers beyond health status.
Model D	Healthcare in country of residence	Mobility pattern, language, education, country group, health outcomes	Tests local healthcare-system anchorage.
Model E	Healthcare in another country	Mobility pattern, language, country group, health outcomes	Tests multi-sited healthcare strategies.

**Table 7:** Variables in the regression models and their conceptual placement.

Variable/construct	Conceptual category	Manuscript use
Current place of residence	Mobility pattern/transnational residence	Main exposure for transnational ageing and healthcare access
Gender	Gender	Intersectional stratifier
Age	Ageing-population stratifier	Captures later working life, transition to retirement and older age
Level of education	Socioeconomic resources	Indicator of life-course resources and vulnerability
Host-country language proficiency	Institutional navigation and agency	Mechanism linking migration status to healthcare access
Main language	Linguistic and cultural background	Marker of linguistic position and potential minority status
Country group	Welfare and regional context	Comparative European context for healthcare access
Healthcare received in Latvia	Healthcare access	Indicator of country-of-origin healthcare reliance
Healthcare received in the country of residence	Healthcare access	Indicator of local healthcare access and welfare anchorage
Healthcare received in another country	Healthcare access	Indicator of third-country or multi-sited healthcare strategy
Poor physical health days in the past 30 days	Physical health outcome	Indicator of recent physical morbidity and active-ageing capacity
Self-rated general health	Physical health outcome	Global indicator of perceived health status
Poor psychological health days in the past 30 days	Psychological wellbeing outcome	Indicator of recent psychological distress
Frequent mental distress	Psychological wellbeing outcome	Indicator of elevated psychological vulnerability
Poor/fair self-rated health	Health vulnerability outcome	Binary outcome for modelling later-life physical health vulnerability
Any poor psychological health days	Psychological wellbeing outcome	Binary outcome for modelling psychological wellbeing differences

Together, these analyses support a coherent interpretation: among ageing Latvian migrants, health burden and healthcare access are related but analytically distinct. Mobility pattern does not significantly differentiate recent poor psychological or physical health days, but it strongly differentiates where care is obtained. This suggests that later-life healthcare use among migrants reflects not only morbidity, but also mobility histories, language, welfare anchorage, trust, cost and the practical ability to navigate health systems across borders.

## 4 Discussion

### 4.1 Main findings

This study examined whether health outcomes and healthcare access differ by mobility pattern among Latvian migrants aged 45 years and older. The findings show a clear distinction between health burden and healthcare-access location. Poor psychological health days and poor physical health days in the past 30 days did not differ significantly by current place of residence. However, healthcare-use location differed substantially by mobility pattern. Respondents who alternated between Latvia and another country were much more likely to have received healthcare in Latvia and less likely to have received healthcare in their country of residence, compared with respondents living outside Latvia. They were also the group most likely to report receiving healthcare in a third country.

This pattern suggests that ageing migrants’ healthcare behaviour cannot be interpreted simply as a function of worse health in one group. Rather, the findings point to the importance of healthcare navigation, language, institutional familiarity, welfare anchorage, trust, cost and transnational mobility. In other words, similar levels of recent physical or psychological health burden may coexist with very different strategies for obtaining healthcare. This distinction is central for understanding active ageing among migrants: opportunities for healthy and secure ageing depend not only on health status, but also on whether older migrants can effectively access care in the systems where they live.

### 4.2 Health outcomes and the meaning of non-significant differences

The absence of statistically significant differences in poor psychological and physical health days by mobility pattern should not be interpreted as evidence that health and wellbeing are unproblematic among ageing Latvian migrants. Instead, it suggests that current place of residence alone is too crude a marker for identifying vulnerability. This is consistent with broader ageing-migration research, which emphasises that older migrants are highly heterogeneous and that vulnerability is shaped by intersecting factors such as gender, socioeconomic position, language, length of residence, legal status, family separation, social support and discrimination (Ciobanu et al., 2017; Smarika et al., 2023).

Recent research on older migrants’ mental health similarly shows that psychological vulnerability in later life often reflects accumulated life-course disadvantage, reduced social networks, physical health decline, migration-related loss and limited access to support systems rather than migration status alone (Anantapong et al., 2024). In this study, mobility pattern did not significantly differentiate the number of poor psychological-health days, but this does not rule out important differences by other factors, including gender, education, host-country language proficiency, economic security, main language or perceived belonging. Future analyses should therefore examine whether psychological distress is more strongly patterned by socioeconomic and linguistic resources than by residence pattern alone.

The same reasoning applies to physical health. Poor physical-health days did not differ significantly across the mobility groups, but this does not mean that physical health is unrelated to migration. Older migrants may have experienced physically demanding work, interrupted employment careers, fragmented access to preventive healthcare, or delayed treatment due to cost, language and administrative barriers. These life-course processes may not be fully captured by current place of residence. For active ageing research, this is important because physical health is not only an outcome; it is also a resource for participation, autonomy and social inclusion in later life.

### 4.3 Healthcare access as the central empirical contrast

The strongest empirical result concerns healthcare access. Healthcare use in Latvia increased markedly across the mobility gradient: from 34.8% among those living outside Latvia to 55.5% among those living mostly outside Latvia and 72.6% among those alternating between Latvia and another country. At the same time, healthcare use in the country of residence decreased from 87.2 to 76.2% and then to 58.7%. This inverse pattern indicates that the more mobile the respondent is in relation to Latvia, the more likely they are to rely on Latvian healthcare and the less likely they are to rely exclusively on the healthcare system of the country of residence.

This finding supports the argument that older and ageing migrants may assemble healthcare across borders. Such healthcare use can be interpreted in two ways. On the one hand, it may represent agency: migrants actively use transnational resources, linguistic familiarity, family support and trusted medical contacts to secure care. On the other hand, it may indicate structural barriers in the country of residence: difficulties with language, digital systems, entitlement rules, waiting times, costs, discrimination, or lack of confidence in local healthcare providers. These interpretations are not mutually exclusive. Transnational healthcare may simultaneously reflect both resourcefulness and exclusion.

This dual interpretation is consistent with research on transnational healthcare among older Russian-speaking migrants in Finland. Shin et al. (2022) show that transnational healthcare use among older migrants is shaped by digital information technology, social integration and perceived discrimination in destination-country healthcare. Their findings suggest that cross-border healthcare is not merely a nostalgic preference for the country of origin, but a practical response to the interaction between local barriers and transnational resources. The Latvian findings point in the same direction: healthcare in Latvia may be chosen because it is linguistically familiar, trusted, affordable or administratively easier, but it may also compensate for weaker access in the host country.

### 4.4 Active ageing, security and welfare anchorage

The results are highly relevant to the WHO active ageing framework, which emphasises health, participation and security as central conditions for quality of life in older age (World Health Organization, 2002). Healthcare access belongs to all three domains. It supports health directly, enables participation by maintaining functional capacity, and contributes to security by reducing uncertainty about illness, treatment and care continuity.

For migrants, however, active ageing may be complicated by the fact that these domains are distributed across more than one country. Some older migrants may live in one country, maintain family or property ties in another, receive pensions from several systems, and use healthcare in different places. This creates opportunities but also risks. Cross-border healthcare may increase flexibility, but it may also fragment medical records, weaken preventive care, delay treatment, and complicate follow-up for chronic disease. In later life, these risks become more serious because health needs often become more complex and continuity of care becomes more important.

The concept of welfare anchorage is useful here. Respondents living outside Latvia and using healthcare mainly in the country of residence appear more anchored in the host-country healthcare system. Respondents alternating between Latvia and another country appear less anchored in one system and more reliant on transnational healthcare arrangements. This does not necessarily mean they are disadvantaged, but it indicates that their active ageing depends on the practical coordination of care across borders. From a policy perspective, the challenge is therefore not only whether older migrants have formal healthcare rights, but whether those rights are usable in practise.

### 4.5 Migration cohort and life-course interpretation

The findings should also be interpreted in relation to Latvia’s heterogeneous migration history. Older Latvian migrants are not one uniform group. People who migrated before or during the Second World War, or who belong to long-established diaspora communities, are likely to differ substantially from migrants who left after 1990 or after Latvia’s accession to the European Union in 2004. Earlier diaspora groups may have spent most of their lives in the host country and may therefore be more institutionally integrated into local healthcare, pension and social-care systems. By contrast, post-1990 and post-2004 migrants may have more fragmented welfare biographies, stronger practical ties to Latvia, and a greater likelihood of transnational healthcare use.

This distinction is important for interpreting the healthcare-access findings. Reliance on healthcare in Latvia among ageing migrants may be especially relevant for those whose migration occurred in adulthood or later working life. These migrants may have retained Latvian-language medical contacts, family support and familiarity with Latvian healthcare institutions. At the same time, they may face weaker host-country language proficiency, incomplete knowledge of local healthcare systems, or uncertainty about entitlements. Therefore, the observed healthcare pattern may reflect not only current mobility, but also age at migration, duration of residence, migration cohort and life-course anchorage.

This interpretation is supported by Lulle’s work on post-socialist active ageing through migration for work. Lulle (2020) argues that migration may reverse conventional assumptions about retirement by enabling older adults from post-socialist contexts to extend working life abroad and manage economic insecurity. Such mobility may support active ageing through continued participation, but it may also expose migrants to fragmented welfare arrangements. The Latvian data fit this broader post-socialist pattern: mobility is not only a demographic condition but also a strategy for managing health, income and social security in later life.

### 4.6 Psychological wellbeing, belonging and ambiguous loss

Although the ANOVA results did not show significant differences in poor psychological-health days by mobility pattern, psychological wellbeing remains a central theme for ageing-migration research. Older migrants may experience loneliness, family separation, reduced social networks, uncertainty about belonging, and loss of continuity between past and present identities. These processes may be particularly relevant for migrants who age across borders and maintain attachments to more than one society.

Recent work on ambiguous loss among ageing migrants emphasises that migration can create unresolved forms of loss related to family separation, identity, belonging and homeland attachment, which may affect psychological wellbeing and care needs in later life (Al-Hamad et al., 2025). This perspective is relevant for the Latvian case because reliance on Latvian healthcare may reflect more than practical access. It may also express trust, cultural familiarity, language comfort and a sense of continuity with earlier life. Such factors are difficult to measure quantitatively, but they may strongly influence how older migrants evaluate care and decide where to seek help.

The findings therefore suggest that future research should combine health-service variables with stronger psychosocial indicators, including loneliness, perceived discrimination, belonging, family proximity, social support, and satisfaction with healthcare communication. In active ageing terms, psychological wellbeing should not be treated only as an individual mental-health outcome. It should also be understood as part of social participation, identity continuity and perceived security in later life.

### 4.7 Language, communication and effective healthcare access

Language is a key mechanism linking migration status to healthcare access. Even when older migrants have formal rights to healthcare, limited host-country language proficiency may restrict effective access. Healthcare encounters require the ability to describe symptoms, understand diagnoses, follow treatment instructions, navigate referrals, communicate with administrative staff, use digital booking systems and understand insurance or payment rules. These demands may be especially challenging in later life, when health needs become more complex.

European research on migrant healthcare has repeatedly shown that language barriers, unfamiliarity with healthcare systems, cultural misunderstandings and administrative complexity can reduce access to appropriate care (Mladovsky et al., 2012; Priebe et al., 2011). In the context of older migrants, these barriers may be amplified by digitalisation, cognitive decline, lower health literacy or dependency on family members for interpretation. The Latvian findings suggest that healthcare use in Latvia may partly compensate for these barriers by allowing migrants to obtain care in a familiar language and institutional context.

However, reliance on country-of-origin care is not a sustainable substitute for accessible local healthcare, particularly for emergency care, chronic disease management and long-term care. Policy responses should therefore focus on language-sensitive healthcare navigation, professional interpreting services, accessible information about entitlements, and support for using digital health systems. These measures are necessary to move from formal entitlement to effective access.

### 4.8 Comparison with other European contexts

The Latvian findings correspond to wider European patterns. In Nordic countries, ageing migrant populations are growing, but research shows that their needs are often insufficiently integrated into mainstream ageing policy. Smarika et al. (2023), for example, argue that ageing migrants in Finland should be analysed through intersectionality and vulnerability rather than through assumptions of homogeneous ageing. Similarly, studies from Sweden indicate that older migrants’ wellbeing cannot be understood only through integration indicators, but must also consider social connectedness, belonging and everyday navigation of institutions.

In Southern Europe, migration, ageing and care are closely connected through transnational family arrangements and care labour. Moré (2021) shows how Ecuadorian care workers in Spain negotiate ageing and care responsibilities across borders. Although the Latvian case differs in historical and regional context, it shares the broader European feature that later life may be organised through multi-sited obligations and resources. Older migrants may be care recipients, care providers, economic supporters and transnational family members at the same time.

The Latvian case contributes specifically to the literature on Central and Eastern European mobility. Unlike long-established postcolonial or post-war labour-migrant groups in Western Europe, many Latvian migrants belong to more recent intra-European mobility cohorts. Their ageing trajectories may therefore be characterised by continued movement, partial settlement and cross-border welfare use. This makes Latvia an important case for understanding how active ageing is changing in the context of free movement, post-socialist restructuring and transnational social protection.

### 4.9 Strengths and limitations

A major strength of this study is its focus on ageing migrants from Latvia, a population that is rarely examined in international ageing and health-services research. The study combines health outcomes with healthcare-access location, allowing a distinction between health burden and care-seeking strategies. The analysis also captures different mobility patterns, making it possible to examine transnational healthcare use rather than assuming that migrants receive care only in the country where they reside.

Several limitations should be acknowledged. First, the analysis is based on survey data and is therefore subject to self-reporting and selection bias. Second, the health outcomes used here measure recent poor psychological and physical health days, which may not fully capture chronic illness, disability, functional limitations or diagnosed mental-health conditions. Third, current place of residence is an imperfect proxy for migration cohort, duration of residence and degree of integration. Future analyses should include year of migration, age at migration, length of residence, citizenship, pension source and employment history. Fourth, healthcare use in Latvia, the country of residence and another country is measured as whether care was received during the past 5 years, but the data do not fully identify the type, frequency, urgency, quality or continuity of care. Finally, the cross-sectional nature of the analysis limits causal interpretation.

This study is based on data collected prior to the COVID-19 pandemic. As such, the findings reflect perceptions and patterns of health and healthcare access before widespread structural changes in health systems and public awareness of health risks. Given that the pandemic may have altered how individuals evaluate their health, especially in transnational contexts, future research should revisit these indicators post-pandemic.

### 4.10 Implications for policy and practise

The findings have several policy implications. First, active ageing policies should recognise older migrants as a heterogeneous population. Long-established diaspora members, post-war migrants, post-1990 migrants and post-2004 EU mobile citizens may have different needs, resources and barriers. Second, healthcare systems should treat language and navigation support as core components of access, not as optional services. Third, cross-border healthcare use should be monitored as a potential indicator of both agency and unmet local need. Fourth, European health systems need better mechanisms for continuity of care across borders, including transferable medical records, clear information about entitlements and support for migrants with fragmented welfare biographies.

For Latvian migrants specifically, the high use of healthcare in Latvia among mobile groups suggests that Latvia remains an important health-resource country for its diaspora. This creates both opportunities and responsibilities. Latvia may play a role in providing culturally and linguistically familiar care for its ageing diaspora, but destination countries must also ensure that older Latvian migrants can access appropriate care locally. Active ageing across borders requires coordination between origin and destination countries, especially as post-1990 and post-2004 migrant cohorts enter older age.

## 5 Conclusion

This study examined health outcomes and healthcare-access patterns among Latvian migrants aged 45 years and older. The findings show that mobility pattern did not significantly differentiate recent poor psychological or physical health days. However, it strongly differentiated where healthcare was obtained. Respondents alternating between Latvia and another country were substantially more likely to use healthcare in Latvia and less likely to use healthcare in the country of residence than respondents living outside Latvia.

These findings shift the analytical focus from health status alone to the conditions under which ageing migrants access care. Healthcare use among older migrants is shaped not only by morbidity, but also by migration cohort, mobility, language, institutional familiarity, trust, cost and welfare anchorage. In the context of active ageing, the key issue is therefore not only whether older migrants are healthy, but whether they can secure understandable, continuous and accessible healthcare as they age across borders.

## Data Availability

Access to the survey microdata is restricted for personal data protection reasons and may be granted only upon submission of a duly justified official request. The use of microdata is limited to scientific, non-commercial purposes. Requests for access to the dataset should be addressed to the Institute of Philosophy and Sociology, University of Latvia at fsi@lu.lv.
